# Cordycepin Increases Nonrapid Eye Movement Sleep via Adenosine Receptors in Rats

**DOI:** 10.1155/2013/840134

**Published:** 2013-04-24

**Authors:** Zhenzhen Hu, Chung-Il Lee, Vikash Kumar Shah, Eun-Hye Oh, Jin-Yi Han, Jae-Ryong Bae, Kinam Lee, Myong-Soo Chong, Jin Tae Hong, Ki-Wan Oh

**Affiliations:** ^1^College of Pharmacy, Chungbuk National University, Cheongju 361-763, Republic of Korea; ^2^Department of Pathophysiology, College of Medicine, Nanchang University, Nanchang, Jiangxi 33006, China; ^3^Research Institute of Veterinary Medicine, Chungbuk National University, Cheongju 361-763, Republic of Korea; ^4^College of Oriental Medicine, Wonkwang University, Iksan 570-749, Republic of Korea

## Abstract

Cordycepin (3′-deoxyadenosine) is a naturally occurring adenosine analogue and one of the bioactive constituents isolated from *Cordyceps militaris/Cordyceps sinensis*, species of the fungal genus *Cordyceps*. It has traditionally been a prized Chinese folk medicine for the human well-being. Because of similarity of chemical structure of adenosine, cordycepin has been focused on the diverse effects of the central nervous systems (CNSs), like sleep regulation. Therefore, this study was undertaken to know whether cordycepin increases the natural sleep in rats, and its effect is mediated by adenosine receptors (ARs). Sleep was recorded using electroencephalogram (EEG) for 4 hours after oral administration of cordycepin in rats. Sleep architecture and EEG power spectra were analyzed. Cordycepin reduced sleep-wake cycles and increased nonrapid eye movement (NREM) sleep. Interestingly, cordycepin increased *θ* (theta) waves power density during NREM sleep. In addition, the protein levels of AR subtypes (A_1_, A_2A,_ and A_2B_) were increased after the administration of cordycepin, especially in the rat hypothalamus which plays an important role in sleep regulation. Therefore, we suggest that cordycepin increases theta waves power density during NREM sleep via nonspecific AR in rats. In addition, this experiment can provide basic evidence that cordycepin may be helpful for sleep-disturbed subjects.

## 1. Introduction

Purines are ubiquitous molecules with important roles in the regulation of metabolic networks and signal transduction events. In the central nervous systems (CNSs), adenosine and ATP modulate the sleep-wake cycles, acting as ligands of specific transmembrane receptors and as allosteric effectors of key intracellular enzymes for brain energy expenditure [1]. Adenosine levels are influenced by neuronal activity. Adenosine is a secondary by-product of the breakdown of ATP and cAMP. When ATP is coreleased with neurotransmitters, ectonucleotidases in the extracellular space can rapidly dephosphorylate ATP, ADP, and AMP into adenosine. ATP release from astrocytes also contributes to extracellular levels of adenosine that have a powerful modulatory effect on synaptic transmission [2]. The role of this astrocyte-derived adenosine in sleep-waking homeostasis was recently investigated. Further research showed that systemic administration of adenosine, its analogs, or inhibitors of its metabolism increase nonrapid eye movement (NREM) sleep in rodents, especially [3].

The local administration of adenosine and/or adenosine receptors (ARs) agonists into the medial preoptic area of hypothalamus, magnocellular cholinergic basal forebrain, brainstem cholinergic areas, the laterodorsal and pedunculopontine tegmental nuclei (LDT/PPT), and pontine reticular formation leads to sleep or reduction of wakefulness [4–6]. In the basal forebrain, both cholinergic and noncholinergic neuronal activities are associated with promoting wakefulness [7]. The somnogenic effects of adenosine may be due to the inhibition of neuronal activity in both cholinergic and noncholinergic neurons of the basal forebrain. In addition, the modulatory effects of sleep deprivation on the A_1_R mRNA in the cholinergic basal forebrain suggest the significance of an adrenergic pathway in the long-term effects of sleep deprivation on the quality of ensuring sleep [8]. Cholinergic neurons of LDT and PPT also comprise the cholinergic “arousal system.” During the transition from waking to sleep, the firing rate of LDT/PPT neurons markedly is decreased, reducing the cholinergic tone of their target sites and thus facilitating the transition to sleep [9]. In particular, the ventrolateral preoptic area of the hypothalamus contains a population of sleep-active neurons and is hypothesized to be an important part of the somnogenic process [10].

The four adenosine forms make up the family of G-protein-coupled AR: A_1_, A_2A_, A_2B_, and A_3_ [11]. The difference between the four ARs is found in their affinity for adenosine, in the type of G proteins that they associate with, and the signaling pathways that are activated in the target cells. For sleep-waking homeostasis, A_1_R and A_2A_R have received the most attention due to their expression pattern in the nervous system, the availability of selective agonists, and antagonists and selective molecular lesions of genes encoding the receptor subtypes. A_1 _and A_3_Rs have high and low affinity for adenosine [12]. Possible changes in adenosine functioning due to the aging process have been observed in animal models, and abnormalities in the adenosine system could also explain primary insomnia or the reduced amount of delta waves sleep and increased sensitivity to caffeine in some subjects with sleep deficits. Caffeine is a methylated derivate of xanthine with profound effects on the onset and quality of sleep episodes [13]. This purine acts principally as an antagonist of the A_2A_R. Adenosine and ATP in the nervous systems are the bridge between metabolic activity, recovery function, and purinergic transmission that underlies the daily wake-sleep cycle in mammals. Modulators of purine actions have the potential to alleviate insomnia and other sleep disorders based on their physiopathological role during the sleep process [14].

**Figure 1:**
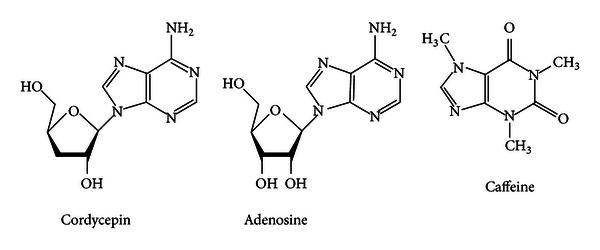
Chemical structures of cordycepin, adenosine, and caffeine.

Cordycepin (3′-deoxyadenosine, Figure 1) is a naturally occurring adenosine analogue and one of the bioactive constituents of *Cordyceps sinensis/militaris* [15, 16]. *C. sinensis/militaris* has been used for hundreds of years as a traditional medicine in treating disorders of the lung and kidney through mechanisms of immunomodulation [17]. Because of similarity of chemical structure of adenosine, cordycepin has been interested in the diverse effects of CNS, like sleep regulation. It also has traditionally been focused on the treatment of insomnia. In particular, cordycepin is known to be a bioactive constituent to regulate homeostatic function [18]. Recently, some herbs have been the charming medicines for considerable sufferers with sleep disabilities or insomnia [19]. Most remedies of sleep aid originated from herbs have been targeted on GABA_A_ systems. However, recent morphological and functional studies have identified AR in the ventrolateral preoptic areas of the hypothalamus that plays an important role in sleep regulation [20]. Therefore, this study was designed to know whether cordycepin increases sleep via AR. This experiment also can provide basic evidence that cordycepin may be helpful for the treatment of insomnia.

## 2. Materials and Methods

### 2.1. Experimental Animals

Male Wistar rats (Samtako, Osan, Korea) weighing 250–300 g were used for sleep recording and western blot. Each rat was housed in each acrylic cage (45 × 60 × 25 cm) with water and food available *ad libitum* under an artificial 12 h light-dark cycle (lights on at 7:00 am) and at constant temperature (22 ± 2°C). To ensure adaptation to the new environment, mice and rats were kept in the departmental holding room for 1 week before testing. This study was performed in accordance with the Chungbuk National University Laboratory Animal Research Center guidelines for the care and use of laboratory animals. 

### 2.2. Experimental Procedure

After 7 days postsurgical recovery, cordycepin (2 and 4 mg/kg in distilled saline) was administered to animals for 5 days once per day. On the 5th day, 1 h after final cordycepin treatment, animals were caged in recording system, and spontaneous sleeping was recorded.

### 2.3. Brain Surgery and EEG Recording

 Each rat was implanted with a transmitter (Data Sciences International, TA11CTA-F40, MN, USA) for recording EEG and activity via telemetry. The body of the transmitter was implanted subcutaneously off the midline and posterior to the scapula and was attached to the skin with 3 sutures for stabilization. Leads from the transmitter led subcutaneously to the skull, and the bare ends were placed in contact with the dura through holes made in the skull (*A*: 2.0 (Bregma), *L*: 1.5; *P*: 7.0 (Bregma), *L*: 1.5 contralateral) [21]. The electrodes were anchored to the skull with screws and dental cement. All surgical procedures were performed stereotaxically under aseptic conditions. Surgical anesthesia was achieved with pentobarbital (50 mg/kg, i.p), and all efforts were made to minimize the suffering of the animals. Telemetric recording of cortical EEG and activity was conducted using procedures similar to previous reports. For the EEG signal, the gain of transmitters was set at −0.5/+0.5 volts per/units × 2, and the raw signals generated from the transmitter were in the range of 0.5–20.0 Hz. The signals were processed by a Data Sciences International analog converter and routed to an AD converter (Eagle PC30, USA) housed in a PC computer. The AD converter digitized the EEG and activity signals at 128 Hz. The digitized data were transferred to the computer and displayed graphically. An online fast Fourier transformation (FFT) was performed on the EEG data at 10 sec intervals during data acquisition (1024 samples) after a Hanning window treatment. The FFT analysis generated power density values from 0.0 to 20.0 Hz at a resolution of 0.5 Hz. The FFT data were further averaged in the range of 0 to 20 Hz for every 10 sec. The sleep data and FFT results were saved to the hard disk every 10 sec for additional offline analysis. Movement of the animal in relation to the telemetry receiver generated transistor-transistor logic (TTL) pulses that were collected and counted as a measure of activity. Cordycepin was administered 1 hour before the EEG recording. Recording began at 9:00 am for 4 h. Seven or eight rats were used in each group.

### 2.4. Analysis of Sleep Architecture

The amounts of time in wakefulness, NREM, and REM sleep were determined from the digitized data at 10 sec intervals using sleep analysis software, *SleepSign 2.1* (KISSEI Comtec Co. Ltd., Matsumoto, Japan). Briefly, the software discriminates wakefulness as high-frequency, low-amplitude EEG. NREM was scored based on the presence of spindles interspersed with slow waves in the EEG. EEG power during REM is significantly reduced in lower frequency *δ*-wave (0.75–4.0 Hz) and increased in the range of *θ*-wave activity (5.0–9.0 Hz, peak at 7.5 Hz). The time spent (min) in NREM, REM, total sleep time (NREM + REM), and numbers of sleep-wake cycle were processed to obtain 4 h period totals for each rat. We further calculated the time of each recording spent in the sleep-wake state (wake, NREM, and REM). The absolute EEG power during wakefulness, NREM, and REM were calculated in 0.5 Hz bins from 0.5 to 20 Hz for the entire 4 h reading of each recording process [22]. Data from seven or eight rats in each group were analyzed.

### 2.5. Membrane Protein Preparation

After deep anesthesia (induced by diethyl ether), animals were decapitated, and the brain was quickly removed and chilled in ice cold saline. Coronal sections were made using a Rodent Brain Matrix (ASI Instruments). This was immediately followed by a 1,200 *μ*m section containing the medial basal hypothalamus, and samples were immediately frozen on dry ice and stored at −80°C [23]. Frozen tissue samples were homogenized in PRO-PREPTM protein extraction solution (iNTRON Biotechnology, Inc.). The homogenate was centrifuged at 15,000 g at 4°C for 20 min, and the supernatant was recovered. A portion of the supernatant was collected to determine the protein concentration and for western blot analysis. The concentration of total protein was determined by a modified Lowry method using bovine serum albumin as a standard. The samples were stored at −20°C.

### 2.6. Western Blot

The total proteins of hypothalamus were loaded in each lane, and sodium dodecyl sulphate polyacrylamide gel electrophoresis (SDS/PAGE) was performed using 12% polyacrylamide gels. Proteins were transferred to PVDF membranes (Amersham Hybond-P, GE Healthcare) using a semidry transfer system. Receptors were detected with the following primary antibodies: rabbit antiadenosine receptor A_1_ (diluted 1 : 1000 in PBS containing 0.5% Tween 20, Abcam), mouse antiadenosine receptor A_2A_ (diluted 1 : 1000 in PBS containing 0.5% Tween 20), and goat antiadenosine receptor A_2B_ (diluted 1 : 1000 in PBS containing 0.5% Tween 20). The following horseradish peroxidase secondary antibodies were used: goat antrabbit IgG (diluted 1 : 5000), rabbit antimouse IgG, and antigoat IgG (1 : 5000). After stripping, membranes were developed with rabbit anti-GAPDH (1 : 1000; Santa Cruz Biotechnology Inc, USA) followed by goat antirabbit IgG to confirm equal protein loading. Immunoreactive bands were developed with a BM Chemiluminescence Detection Kit (Roche Diagnostics). Quantitative analysis of detected bands was performed with densitometric scanning, and all values were normalized using GAPDH as a standard [24]. 

### 2.7. Statistical Analysis

All data were analyzed using SPSS 17.0 software (SPSS). Significant differences after one-way ANOVAs were measured by post hoc Holm-Sidak test. *P* < 0.05 was considered to be significant. The values are expressed as mean ± SEM. All statistical analyses were conducted using *SigmaStat* software. 

## 3. Results 

### 3.1. Effects of Cordycepin on the Number of Sleep-Wake Cycles

**Figure 2:**
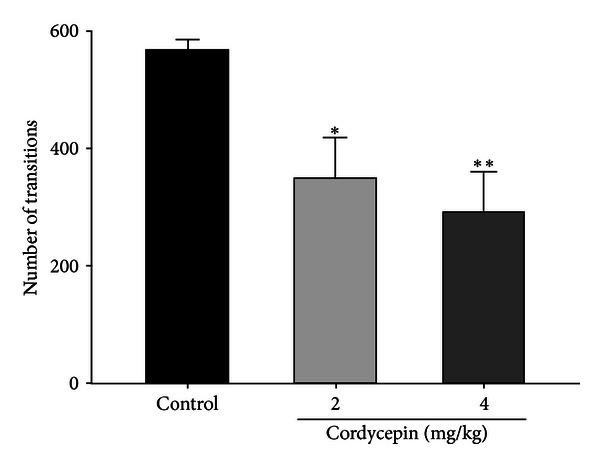
Effects of cordycepin (2 and 4 mg/kg) on sleep-wake cycles. Values are expressed as the mean ± SEM. *P < 0.05 and **P < 0.01, compared with that of the control. Five to six animals were used in each group.

Cordycepin (2 mg/kg, *P* < 0.05) and (4 mg/kg, *P* < 0.01) significantly reduced the number of sleep-wake cycles, respectively, compared with that of the control (Figure 2). 

### 3.2. Effects of Cordycepin on Sleep Architecture

**Figure 3:**
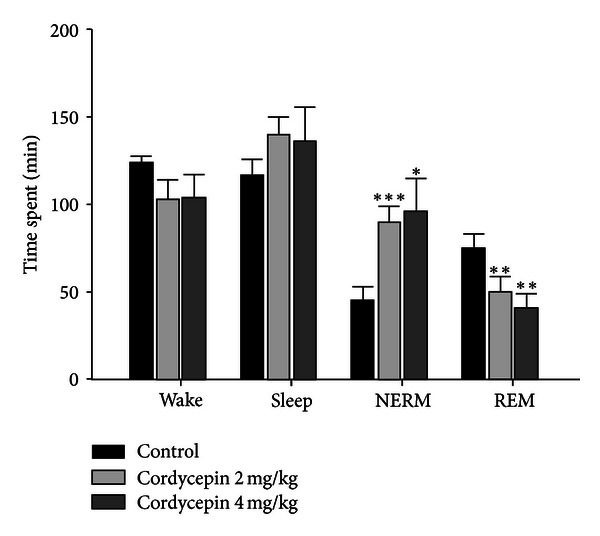
Effects of cordycepin (2 and 4 mg/kg) on rat sleep architecture. Values are expressed as the mean ± SEM. NREM, nonrapid eye movement; REM, rapid eye movement. *P < 0.05, **P < 0.01, and ***P < 0.005, compared with that of the control. For more details, refer Figure 1.

Cordycepin (2 and 4 mg/kg) significantly increased NREM (*P* < 0.005 and *P* < 0.05) and decreased REM sleep (*P* < 0.01 and *P* < 0.01). However, both wakefulness and total sleep time were not changed significantly, compared with that of the control (Figure 3). 

### 3.3. Effects of Cordycepin on EEG Power Density during Total Sleep Time

**Figure 4:**
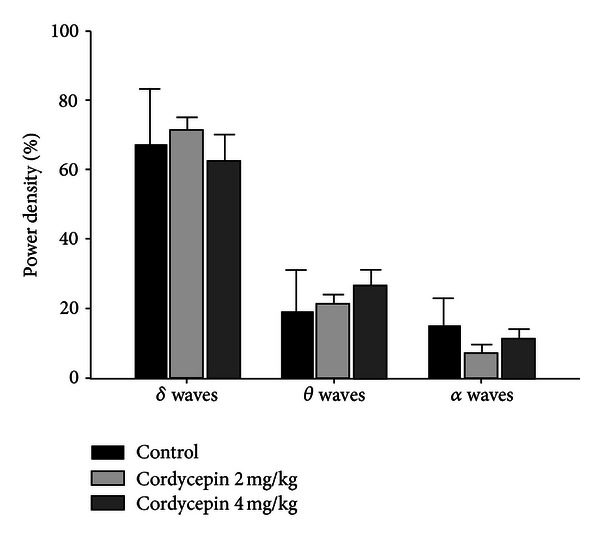
Effects of cordycepin (2 and 4 mg/kg) treatment on EEG power density during total sleep time. EEG power densities in delta wave, theta wave and alpha wave, spectral bandwidths were evaluated during total sleep time. No significant differences, compared with that of the control. For more details, refer Figure 1.

No significant changes in delta wave, theta wave, and alpha waves power density during total sleep time were observed; cordycepin- (2 and 4 mg/kg) treated groups, compared with that of the control (Figure 4).

### 3.4. Effects of Cordycepin on EEG Power Density during NREM Sleep

**Figure 5:**
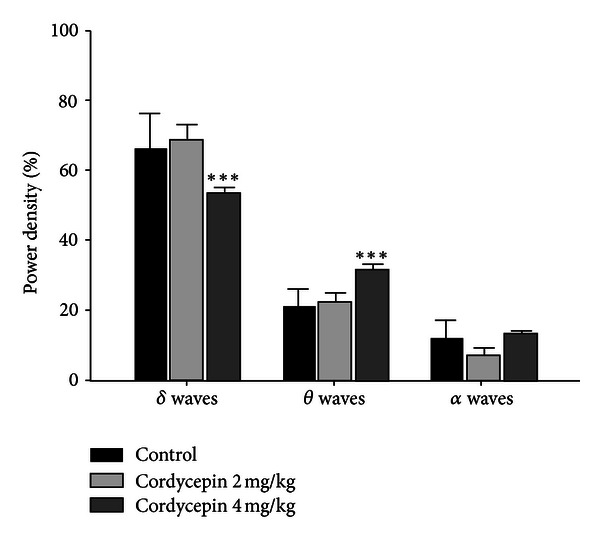
Effects of cordycepin (2 and 4 mg/kg) on EEG power density during NREM sleep. EEG power densities in delta wave, theta wave, and alpha wave spectral bandwidths were evaluated. Values are expressed as the mean ± SEM of EEG power densities in three selected frequency bands during NREM sleep. ***P < 0.005, compared with that of the control. For more details, refer Figure 1.

Interestingly, cordycepin decreased delta waves (*P* < 0.005) during NREM sleep. However, theta waves (*P* < 0.005) during NREM sleep significantly were increased by cordycepin (4 mg/kg). No changes in alpha waves power density during NREM sleep were observed, compared with that of control (Figure 5).

### 3.5. Effects of Cordycepin on EEG Power Density during REM Sleep

**Figure 6:**
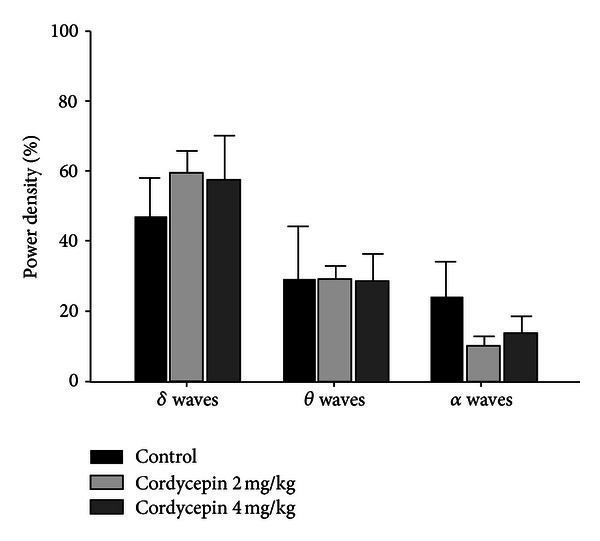
Effects of cordycepin (2 and 4 mg/kg) on EEG power density during REM sleep. EEG power densities in delta wave, theta wave, and alpha wave spectral bandwidths were evaluated. Values are expressed as the mean ± SEM of EEG power densities in three selected frequency bands for the REM sleep. No significant differences, compared with that of the control. Five to six animals were used in each group. For more details, refer Figure 1.

No significant changes were observed in delta wave, theta wave, or alpha wave power density during REM sleep in cordycepin- (2 and 4 mg/kg) treated groups, compared with that of the control (Figure 6).

### 3.6. Effects of Cordycepin on the Protein Levels of the A_1_R, A_2A_R, and A_2B_R Subtypes in the Rat Hypothalamus

**Figure 7:**
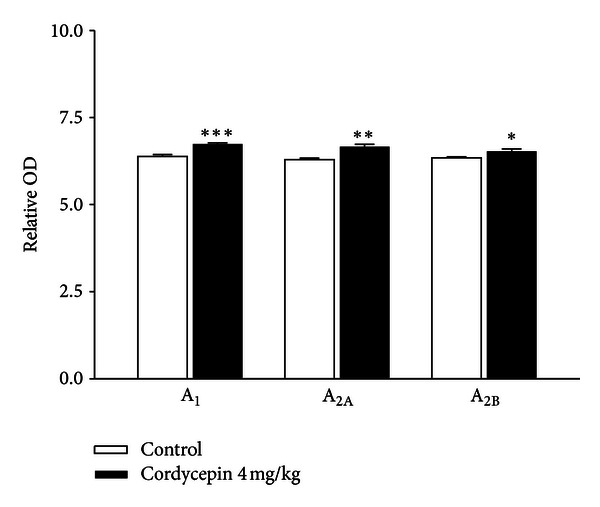
Expression of AR: A_1_, A_2A_, and A_2B_ in the rat hypothalamus after cordycepin treatment. AR subtypes in the rat hypothalamus after cordycepin treatment were analyzed by western blotting. The intensity of the immunoreactive bands of 3 to 4 independent experiments was measured by densitometry scanning and normalized using glyceraldehyde 3-phosphate dehydrogenase (GAPDH) as a standard (bar graph). Results are presented as the percentage immunoreactivity detected in the hypothalamus with respect to the GAPDH protein loading control. Values are expressed as the mean ± SEM. *P < 0.05, **P < 0.01, and ***P < 0.005, compared with that of the control.

The protein levels of the A_1_R, A_2A_R, and A_2B_R subtypes in the hypothalamus were measured by western blot after codycepin administration. *Cordyceps* (4 mg/kg) increased the protein levels of A_1_R, A_2A_R, and A_2B_R subtypes (***P < 0.005, **P < 0.01, and *P < 0.05), compared with that of the control (Figure 7). 

## 4. Discussion

*C. sinensis/militaris* which contain cordycepin (3′-deoxyadenosine), a naturally occurring adenosine analogue and one of the bioactive constituents, have been used for hundreds of years as traditional medicines in treating insomnia [15]. In this study, cordycepin reduced sleep-wake cycles, increased NREM sleep, and decreased REM sleep. It also increased total sleep time in rodents and decreased wakefulness. Interestingly, power spectral analysis showed that cordycepin significantly increased theta waves power density during NREM sleep. Therefore, it will be suggested that cordycepin, an adenosine analogue, plays a role in the modulation of theta oscillations in NREM sleep. Sleep in most mammals is divided into two major types of sleep, REM sleep and NREM sleep. REM sleep is characterized by fast waves sleep with muscle atonia, activation of brain, and eye movement. NREM sleep characterized by slow waves sleep is emerged in delta waves [25]. During NREM sleep, neuronal activity, metabolic rate, and brain temperature are low. From the EEG experiment, cordycepin increased NREM sleep and reduced REM sleep. However, it tended to increase slow waves in total sleep although they were not significantly increased. On the contrary, theta waves in NREM sleep were significantly increased, delta waves in NREM sleep slightly decreased, and no change in alpha waves was observed. Theta waves between alpha and delta waves in wavelength specially were increased in NREM sleep [26]. 

Multiple interacting neurotransmitter systems in the brain stem, hypothalamus, and basal forebrain converge onto common effector systems in the thalamus and cortex. Sleep results from the inhibition of wake-promoting systems by homeostatic sleep factors such as adenosine, nitric oxide, and GABAergic neurons in the preoptic area of the hypothalamus, resulting in large-amplitude, slow EEG oscillations [27]. One ubiquitous neuromodulatory system in the CNS is operated by adenosine. Adenosine is an important homeostatic sleep factor acting in basal forebrain and preoptic areas through A_1_R and A_2A_R. Recent morphological and functional studies have identified AR in the ventrolateral preoptic areas of the hypothalamus that plays an important role in sleep regulation [28]. Adenosine neuromodulation is mostly conceived as an inhibitory system that restrains excitatory transmission through activation of inhibitory A_1_R. Furthermore, adenosine modulates the neuronal activity of basal forebrain and preoptic/anterior hypothalamic in the control of behavioral state [7]. The sleep-inducing effect of adenosine is most efficient when adenosine or its agonists are applied locally into the basal forebrain and preoptic/anterior hypothalamus [29, 30]. The compensatory sleep response to sleep deprivation is significantly reduced when the increase in adenosine or A_2_R activation is blocked [31]. A_2A_R is expressed in the brain areas such as striatum, nucleus accumbens, and olfactory tubercle [32]. Selective A_2A_R agonist administration into the subarachnoid space adjacent to the basal forebrain and the lateral preoptic area indduces NREM sleep [33]. In contrast to other brain areas, increases in extracellular adenosine levels in the basal forebrain are very sensitive to even short periods of prolonged waking; adenosine levels continue to rise throughout the waking period and do not decline until sleep is initiated. As the basal forebrain is essential for wake promotion, the sleep-inducing effect is thought to be mediated via inhibition of the wake-active basal forebrain cells [34, 35]. Prolonged waking activates inducible nitric oxide synthase in the basal forebrain, which causes adenosine release and recovery sleep through energy depletion. There are numerous reports indicating that endogenous adenosine is a candidate for the homeostatic sleep factor theory inducting sleep after prolonged wakefulness [35]. 

Adenosine's pivotal role in sleep modulation is strongly supported by the subjective and EEG-defined arousal produced by its antagonists, caffeine, and theophylline, as well as by the fact that extracellular adenosine concentration is linked to neuronal metabolic activity [1, 36]. Researches showed that systemic or local administration of adenosine, its analogs, or inhibitors of its metabolism increase especially NREM sleep in rodents [37]. The mechanism by which cordycepin increased NREM sleep and decreased wakefulness was probably achieved through activation of adenosine receptors. Thereby, we suggest that cordycepin mediated effects on sleep-wake states are site and receptor dependent. Furthermore, cordycepin may pharmacologically act in the same manner as that of adenosine as cordycepin has affinity at the A_1_, A_2_, and A_3_ receptors [38]. For sleep-waking homeostasis, A_1_R and A_2A_R have received the most attention due to their expression pattern in the nervous system. A_1_R is widely distributed in CNS and inhibits especially cholinergic neurons from forebrain and LDT/PPT [39]. Stimulation of A_1_R results in adenylate cyclase inhibition and phospholipase C activation. Higher level of A_1_R agonist or higher expression of A_1_R potentiates the phospholipase C pathway [40]. The activation of presynaptic A_1_R inhibits neurotransmitter release, mostly of excitatory neurotransmitter such as glutamate and acetylcholine. From this study, cordycepin activated AR subtypes. So the present report strongly suggests that cordycepin enhances NREM sleep in activating upon AR. 

Therefore, administration of cordycepin, an adenosine analogue, widely modulated the power spectral densities of EEG architecture. Increase of total sleep and NREM sleep by cordycepin might be related to the action upon AR. Moreover, the mechanisms which cordycepin modulates EEG architecture and sleep behaviors may be attributed to the similar chemical structure of adenosine. The use of several herbs in the treatment of sleep disorder, such as insomnia, is regarded as a developing market for agents acting on GABA receptors. St. John's Wort, valerian extracts, and ginseng species are the best examples of this issue [41–43]. In addition, ginseng extract enhanced the total sleep and NREM sleep, and reduced the number of sleep-wake cycles [44, 45]. Nevertheless, this is the first experiment that cordycepin, a natural product for the treatment of sleep disorders, such as insomnia, acts on AR. 

Cordycepin is quickly deaminated by adenosine deaminase and rapidly metabolized to an inactive metabolite, 3′-deoxyhypoxanthinosine, in vivo [46]. Adenosine is otherwise transported into cells by several nucleoside permeases, and then the adenosine will be phosphorylated to form ATP by adenosine kinase. If the cell does not utilize the adenosine, it will be deaminated to hypoxanthinosine by adenosine deaminase inside the cell [47]. So adenosine and cordycepin had short elimination half-lives and high rates of clearance [18]. We also presume that cordycepin has short-acting compound in the regulation of sleep. Therefore, EEG recording was performed for 4 hours. 

## 5. Conclusions

Cordycepin, targeting on AR, can be helpful for sleep disturbance subjects. Thus, the results also indicated a role for cordycepin, an adenosine analogue, in the regulation of sleep and held promise for a new class of compounds as potential agents in the treatment of sleep disorders, such as insomnia. Cordycepin as an adenosine analogue increased NREM sleep and decreased REM sleep, suppressing waking. Cordycepin also increases theta waves power density during NREM sleep via nonspecific AR in the ventrolateral preoptic area of the hypothalamus of the rats. Further research would be necessary to understand the cellular and molecular mechanisms of cordycepin-induced sleep modulation in the brain.
